# Visibility-Graph Analysis of Raman Spectra in Chalazion and Lipid Dysregulation: An Exploratory Study

**DOI:** 10.3390/e28091011

**Published:** 2026-09-11

**Authors:** Renata Wojnarowska-Nowak, Rafał Rak, Kornelia Łach, Aneta Kowal, Kamil Szmuc, Damian Roczkowski, Rafał Kuna, Kamila Nester-Ostrowska, Zozan Guleken, Józef Cebulski, Aneta Lewicka-Chomont

**Affiliations:** 1 Institute of Materials Engineering, Faculty of Exact and Technical Sciences, University of Rzeszow, Pigonia 1, 35-310 Rzeszów, Poland; rwojnarowska@ur.edu.pl (R.W.-N.); kszmuc@ur.edu.pl (K.S.); 2 Institute of Physics, Faculty of Exact and Technical Sciences, University of Rzeszow, Pigonia 1, 35-310 Rzeszów, Poland; droczkowski99@gmail.com (D.R.); jcebulski@ur.edu.pl (J.C.); 3 Department of Medical Chemistry and Metabolomics, Faculty of Medicine, Medical College, University of Rzeszow, al. T. Rejtana 16c, 35-959 Rzeszów, Poland; kolach@ur.edu.pl; 4 Doctoral School, Institute of Medical Sciences, Medical College, University of Rzeszow, al. T. Rejtana 16c, 35-959 Rzeszów, Poland; anekowal@ur.edu.pl; 5 Department of Semiconductor Materials Engineering, Wroclaw University of Science and Technology, Wybrzeże Wyspiańskiego 27, 50-370 Wrocław, Poland; rafal.kuna1983@gmail.com; 6 Laboratory of Ophthalmology, Faculty of Medicine, Medical College, University of Rzeszow, al. T. Rejtana 16c, 35-959 Rzeszow, Poland; knester@ur.edu.pl (K.N.-O.); alewicka@ur.edu.pl (A.L.-C.); 7 Ophthalmology Clinic, University Clinical Hospital, Szopena 2, 35-055 Rzeszow, Poland; 8 Department of Physiology, Faculty of Medicine, Gaziantep Islam Science and Technology University, Gaziantep 27010, Türkiye; zozanguleken@gmail.com

**Keywords:** graph-based signal analysis, chalazion, human serum, lipid disorders, Raman spectroscopy, principal component analysis, visibility-graph analysis

## Abstract

A chalazion is an inflammatory lesion of the eyelid caused by a blockage in the sebaceous glands. To identify subtle biochemical differences in chalazion secretions and patient serum, we apply a graph-theory-based visibility-graph approach to Raman spectra, focusing on its exploratory potential for detecting band-specific spectral differences. As a classical baseline, we also employ Principal Component Analysis (PCA); however, the visibility-graph approach provides complementary, band-localized information that may support interpretation of subtle spectral differences. The study included samples from chalazion patients and control subjects, comprising healthy individuals and patients with lipid disorders. Raman spectra were analyzed by transforming the data into visibility graphs, enabling identification of hidden correlations and signal dynamics. Within the VG representation, group differences were concentrated mainly in the 1100–1300 cm^−1^ range, with localized extrema near 1177, 1184, and 1260 cm^−1^ in chalazion secretions and near 1172, 1174, 1219, and 1257 cm^−1^ in serum. The identified positions overlap regions with possible lipid- and protein-related contributions, including C–C, =CH, and amide III vibrations, and are treated as candidate spectral positions rather than markers of individual molecular compounds. From the perspective of complex-network-based signal analysis, the visibility-graph transformation provides an alternative way of representing Raman spectra and extracting structural information that is not readily apparent in the original spectral domain. This information-oriented approach is consistent with entropy-related studies of complex signals, where nonlinear structure, local organization, and hidden dependencies in data are of central interest. These findings suggest that visibility-graph analysis may be useful as an exploratory tool for identifying candidate lipid-related spectral features, although further validation using larger sample sets is required. Full-range PCA was also examined over 200–2000 cm^−1^. It revealed global covariance patterns distributed across several spectral regions, whereas VG localized the pairwise differences within the 1100–1300 cm^−1^ range at specific Raman-shift positions.

## 1. Introduction

The meibomian glands secrete meibum, which is a complex mixture of different lipids that plays an important role in tear film stability and in preventing evaporation [1]. Chalazion is an inflammatory lesion that forms when lipid breakdown products leak into surrounding tissue and trigger an inflammatory response. Chalazion can be classified as deep or superficial: a deep chalazion is caused by inflammation of a tarsal meibomian gland, whereas a superficial chalazion results from inflammation of a Zeis gland. In most cases, chalazion is a benign condition; however, antibiotic and steroid therapy may be required, and in some cases, surgical intervention is necessary [2,3].

Even though chalazion is one of the most prevalent eyelid conditions across all age groups, the current literature contains inconsistent pathophysiological, epidemiological, and therapeutic data [4]. Recent studies have linked changes in lipid profiles of meibomian gland secretions to systemic conditions, such as dyslipidemia [5]. Furthermore, changes in meibomian gland function and morphology have been shown in patients with lipid disorders, as evidenced by imaging techniques such as infrared meibography [6].

Analysis of the composition of the chalazion using high-performance liquid chromatography/mass spectrometry (HPLC/MS) revealed the presence of very long- and extremely long-chain wax esters; free (non-esterified) cholesterol; cholesteryl esters, including cholesteryl esters of (O-acyl)-ω-hydroxy fatty acids; triacylglycerols; ceramides; phosphatidylcholines; and sphingomyelins. The lipid composition was variable and depended on the duration of the lesion [7,8].

Raman spectroscopy is a powerful analytical technique for examining biological samples; specific spectral patterns allow the identification of substances and molecular changes with high specificity. It can easily detect all major biological sample components including lipids, proteins, and nucleic acids, which are crucial for understanding meibomian gland dysfunction and its implications in ocular health [9]. Raman spectroscopy is well suited to the analysis of biological materials because it requires limited sample preparation and is generally non-destructive to the measured sample [10,11]. However, the invasiveness of a clinical procedure depends on the method used to collect the biological material. In the present study, Raman measurements were performed ex vivo on chalazion secretion obtained during surgical curettage and on serum prepared from blood samples collected during routine clinical testing or blood-donor health evaluation. Its successful application in ophthalmology, including the biochemical characterization of ocular tissues and fluids as well as the identification of disease-related molecular alterations, highlights the technique’s versatility and promise for clinical diagnostics [12,13,14].

Recent developments in biomedical sensing also include engineered SERS substrates designed to enhance weak Raman signals and highly sensitive graphene field-effect transistor platforms for biological detection and real-time monitoring [15,16,17]. Although these approaches differ from the conventional Raman microspectroscopy used in the present study, they show the growing role of advanced physical measurements and signal analysis in biomedical research.

In this paper, we report a study of the Raman spectra of the chalazion secretion and serum from patients with chalazion. A detailed analysis of Raman spectra of chalazion secretions is presented, providing information on the composition of the analyzed material and the presence of specific molecules in ocular secretions. To deepen the analysis, statistical techniques were also used: the visibility algorithm (Visibility Graph (VG) framework) and standard PCA. Visibility-graph methods have previously been explored in ophthalmic image analysis, including eye disease images and diabetic retinopathy fundus images [18,19]. However, these applications concerned imaging data rather than vibrational spectra. Therefore, the present study should be understood as an exploratory application of VG analysis to Raman spectra of chalazion secretions and serum, rather than as a clinically validated diagnostic method. The analytical sequence used in the present study was to examine the broad spectral range with VG, compare the resulting localization with full-range PCA, and then examine PCA in the 1100–1300 cm^−1^ range indicated by the VG analysis.

## 2. Materials and Methods

### 2.1. Subjects

The samples were collected from patients at the Ophthalmology Clinic of the University Clinical Hospital No. 1 in Rzeszow and Regional Blood Donor Centre in Rzeszow (Poland), with informed consent. Written informed consent for participation in the study was obtained from the parents and/or legal guardians of all minor participants prior to enrollment. Where appropriate, assent was also obtained from the minors in accordance with applicable ethical guidelines. The study was approved with No. 011/02/2025 by the Bioethics Committee of the University of Rzeszów dated 05/02/2025 to Annex No. 2025/01/008 dated 8 January 2025 to Resolution 5/11/2021 dated 3 November 2021. The study was conducted in accordance with relevant guidelines and regulations.

The study included patients aged 14 to 72 years. Chalazion material was obtained from 7 patients without lipid disorders and compared with 9 patients with lipid disorders. In addition, analyses of serum were performed of patients with chalazions (with and without lipid disorders: 4/4). The control group (K) for serum analysis was three healthy men (honorary blood donors). More detailed patient characteristics are presented in Table 1.

For clarity, two related but distinct sample sets were analyzed. The first set consisted of chalazion secretions obtained from patients without lipid disorders and patients with lipid disorders. The second set consisted of serum samples from chalazion patients without lipid disorders, chalazion patients with lipid disorders, and healthy controls. Therefore, the control group was available only for serum analysis and not for chalazion secretion analysis. As a result of the clinical evaluation, chalazion was diagnosed, and patients were selected for surgical treatment after the inflammation had subsided. During surgery, chalazion secretions were obtained by making an incision in the tarsal conjunctiva of the upper or lower eyelid under local anesthesia, followed by curettage of the contents of the lesion. The collected material was placed in sterile containers, frozen at −80 °C and stored for further research.

The present cohort was designed as an exploratory and feasibility-oriented dataset rather than as a confirmatory diagnostic cohort. Accordingly, the number of independent biological subjects was not considered sufficient for population-level inference, adjustment for age, sex, medication use, lesion duration, or other potential confounding factors. Sample qualification prioritized analytical suitability and pre-analytical consistency. Material showing substantial blood or tissue admixture, fibrotic content, or altered structure that could compromise the comparability of Raman measurements was excluded. These quality-control criteria reduced avoidable pre-analytical heterogeneity but did not compensate for the limited cohort size.

The healthy control group was available only for serum analysis and consisted of three male donors. Therefore, serum comparisons involving group K should be interpreted as descriptive. In addition, because no healthy chalazion-secretion control could be defined, the secretion analysis compares lipid-disorder status among patients with chalazion and cannot independently separate spectral effects associated with chalazion from those associated with systemic lipid dysregulation.

In addition to the analysis of chalazion secretions, serum samples from patients with chalazions, both with and without lipid disorders, as well as from healthy control, were also examined. All serum samples were collected as part of standard medical testing, as an element of diagnostics and treatment of patients with chalazion and health evaluation analysis of honorary blood donors. The serum samples were prepared by allowing the blood to clot at room temperature for 30–120 min, followed by two centrifugations: first at 3000 rpm for 5 min, and then at 5000 rpm for 5 min. The obtained serum samples were frozen at −80 °C until further analysis.

### 2.2. Raman Spectroscopy

Before Raman measurements, frozen chalazion secretion and serum samples were thawed. Both types of samples were deposited on high-quality polished quartz disc substrates (Ted Pella, Redding, CA, USA) and dried at room temperature. Chalazion secretion was applied to the substrate using a microspatula, (Bochem Instrumente GmbH, Weilburg, Germany) whereas serum was applied using an automatic pipette (Corning HTL SA, Warsaw, Poland). The amount of material applied was kept consistent within each sample category. Raman spectra were acquired directly from the dried sample surface; the laser beam interacted directly with the sample rather than through a container or holder. No coverslips were used. All chalazion-secretion samples and all serum samples from groups A, B, and K were prepared and measured according to the same procedure within the corresponding biological matrix.

The Raman spectra were obtained using an InVia Micro Raman Renishaw spectrometer (Renishaw, Wotton-under-Edge, UK) combined with a Leica DM 2500M microscope and a 785 nm laser. Spectra were acquired with an exposure time of 10 s using three accumulations per spectrum for chalazion secretion samples and five accumulations per spectrum for serum samples. The laser output power was set to 5 mW for chalazion secretions and 10 mW for serum. The measurement was carried out using a 50× lens magnification. Data preprocessing included baseline correction (Intelligent polynomial baseline subtraction algorithm), spectral smoothing (Savitzky–Golay method) and normalization using the min-max approach [20,21].

The uncorrected spectra showed an elevated broad background before baseline correction, consistent with luminescence contributions from the biological material. Baseline correction was therefore an important preprocessing step for the Raman intensity data.

To ensure methodological consistency, samples within each biological matrix were handled, stored, preprocessed, and analyzed using the same procedures, and Raman spectra were acquired using the same instrumental settings within each matrix. Chalazion secretion and serum were analyzed as separate datasets because they represent different biological matrices with different biochemical compositions and optical properties. Separate fixed acquisition settings were therefore used for the two matrices.

### 2.3. Data Analysis

#### 2.3.1. Lipid Profile of the Participants

**Figure 1:**
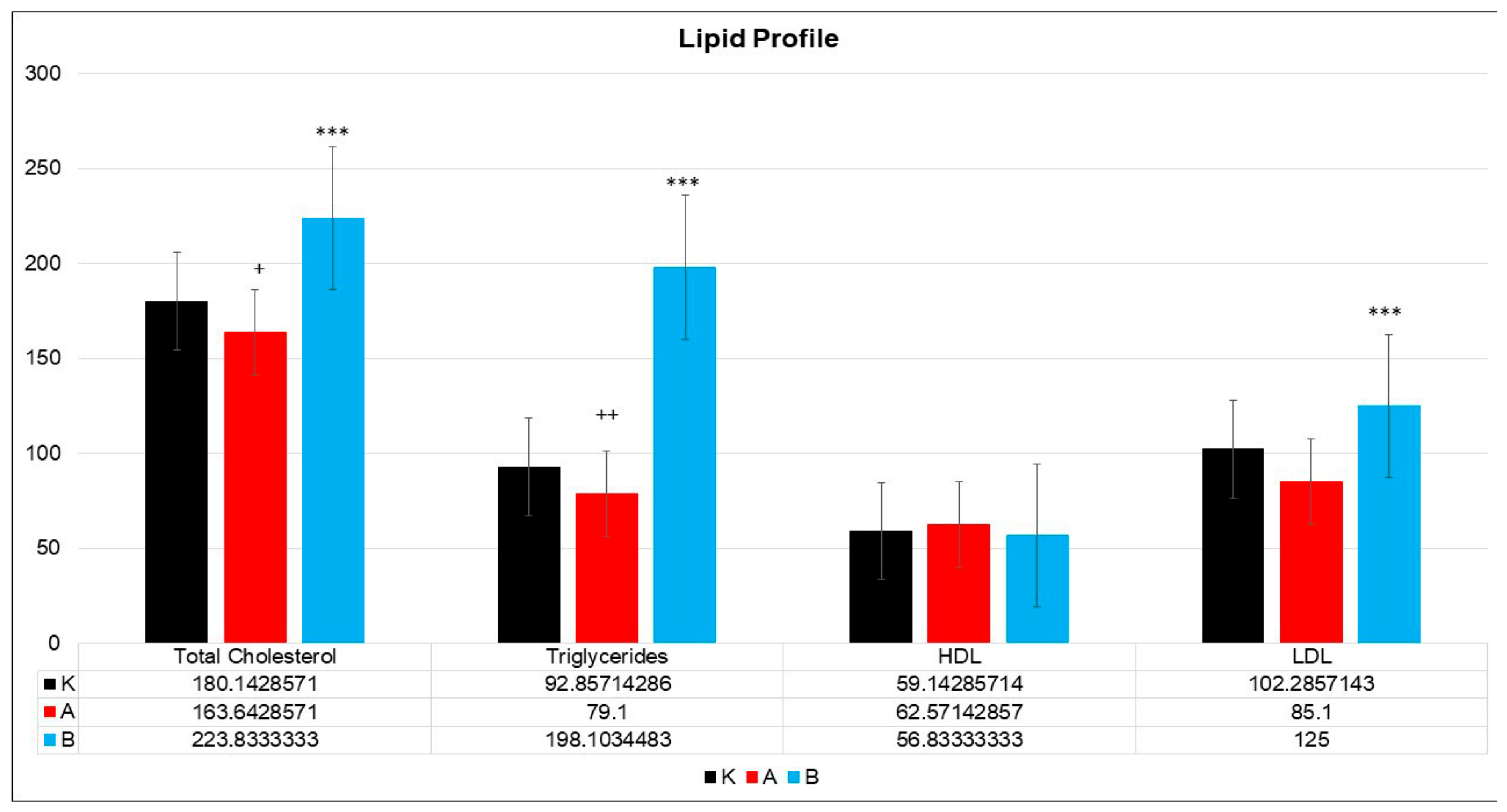
Serum lipid levels in the control group (K), patients with chalazion without lipid disorders (A), and patients with chalazion and lipid disorders (B). ‘+’ denotes comparison with the control group and ‘*’ denotes comparison with group A. ^+^ *p* < 0.05, ^++^ *p* < 0.01 compared with the control group (K); *** *p* < 0.001 compared with the chalazion group without lipid disorders (A).

We measured serum total cholesterol, triglycerides, High-density lipoprotein (HDL) and low-density lipoprotein (LDL) in control subjects (K), patients with chalazion without lipid disorders (A), and chalazion patients with lipid disorders (B) (Figure 1). Group B showed significantly higher total cholesterol and triglyceride levels compared to both the control group (*p* < 0.05) and group A (*p* < 0.001). HDL levels were slightly higher in group A, while LDL levels were highest in group B (*p* < 0.001).

#### 2.3.2. PCA

Principal Component Analysis (PCA) was employed as a classical approach for this type of data [22]. In general, PCA reduces data dimensionality by projecting the original variables onto orthogonal components (PCs) ordered by decreasing explained variance. Visualization was carried out in the PC1–PC2 plane, including 95% covariance ellipses and class centroids. The percentage values shown on the PC1 and PC2 axes denote the proportion of total variance explained by the corresponding component—i.e., the component’s own variance relative to the total variance (computed in practice from eigenvalues/squared singular values). A larger variance contribution on PC1 indicates that a greater proportion of the overall variability is captured along the primary direction of data spread, whereas a substantial contribution of PC2 reflects variability in a direction orthogonal to PC1. Accordingly, a clear displacement of class centroids along the axis with the higher explained-variance percentage generally provides stronger support for group separation. Multiple Raman spectra were recorded from each biological sample at different measurement sites. These spectra were retained to describe local spectral variability within the analyzed material. They were treated as technical measurements and not as independent biological replicates. Therefore, the PCA plots and the 95% covariance ellipses describe the distribution of spectra at the measurement level. They do not represent patient-level confidence regions and do not provide diagnostic validation. The number of technical spectra was not used to increase the number of independent biological subjects.

PCA was evaluated over the full 200–2000 cm^−1^ range and in the 1100–1300 cm^−1^ range. The same preprocessed spectra were used at both spectral scales; no additional preprocessing was introduced for the full-range PCA. Full-range PC1–PC2 score distributions with 95% covariance ellipses and the corresponding PC1 and PC2 loading profiles were evaluated to characterize the global covariance structure of the spectra.

PCA was also examined in the 1100–1300 cm^−1^ window because the VG profiles localized the clearest pairwise differences in this region. This range contains contributions from lipid-related C–C and =CH vibrations and protein-related signals, including amide III [23,24,25]. The same range was used to compare PCA with the spectral localization provided by VG. PC1 and PC2 loadings were calculated for the full 200–2000 cm^−1^ range and for the 1100–1300 cm^−1^ range.

#### 2.3.3. Visibility Algorithm

The concept of ‘visibility’ comes from the complex networks (graphs) theory and is part of a field known as ‘visibility graphs’ (VG) methodology [26,27]. Consider a series, denoted by *x_i_* values with *i* = {1, 2, …, *N*}, where *N* denotes the total number of records. Two arbitrary points *x_a_* and *x_b_*, are visible to each other if all points between them satisfy:

$$x_{c}\leq x_{b}+\left(x_{a}-x_{b}\right)\cdot \frac{b-c}{b-a}\;$$

**Figure 2:**
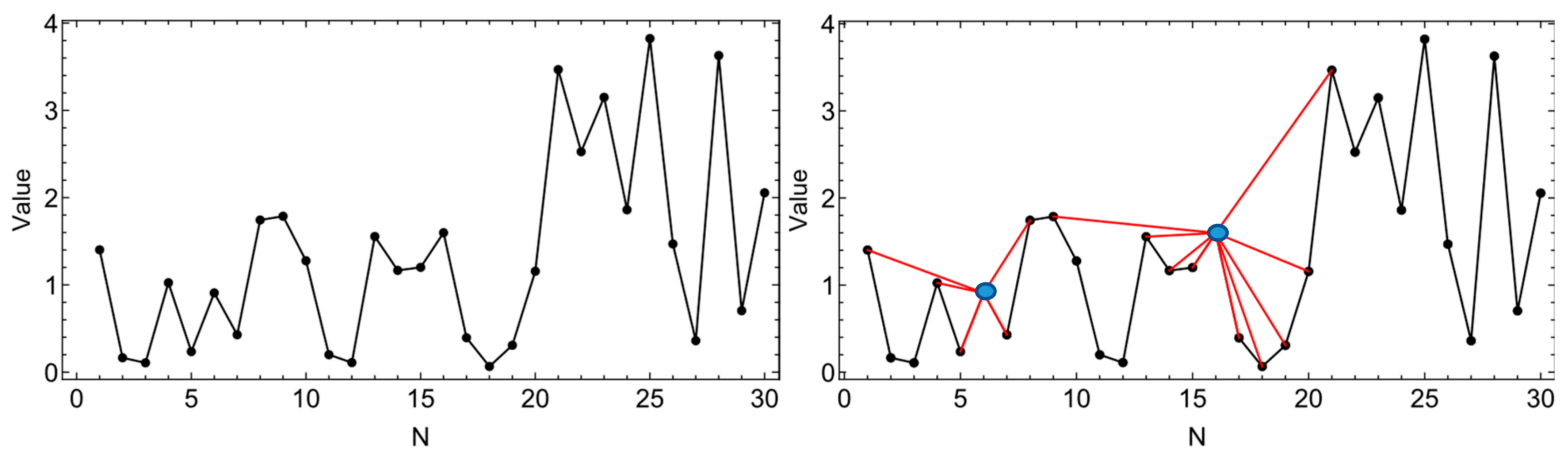
Example data series (**left panel**) and visualization of visibility criterion for two selected points (**right panel**).

Therefore, two points can see each other if no other point obstructs the straight line connecting these two points. Moreover, visibility values are natural numbers. An example data series for N=30 is shown in Figure 2 (left panel).

**Figure 3:**
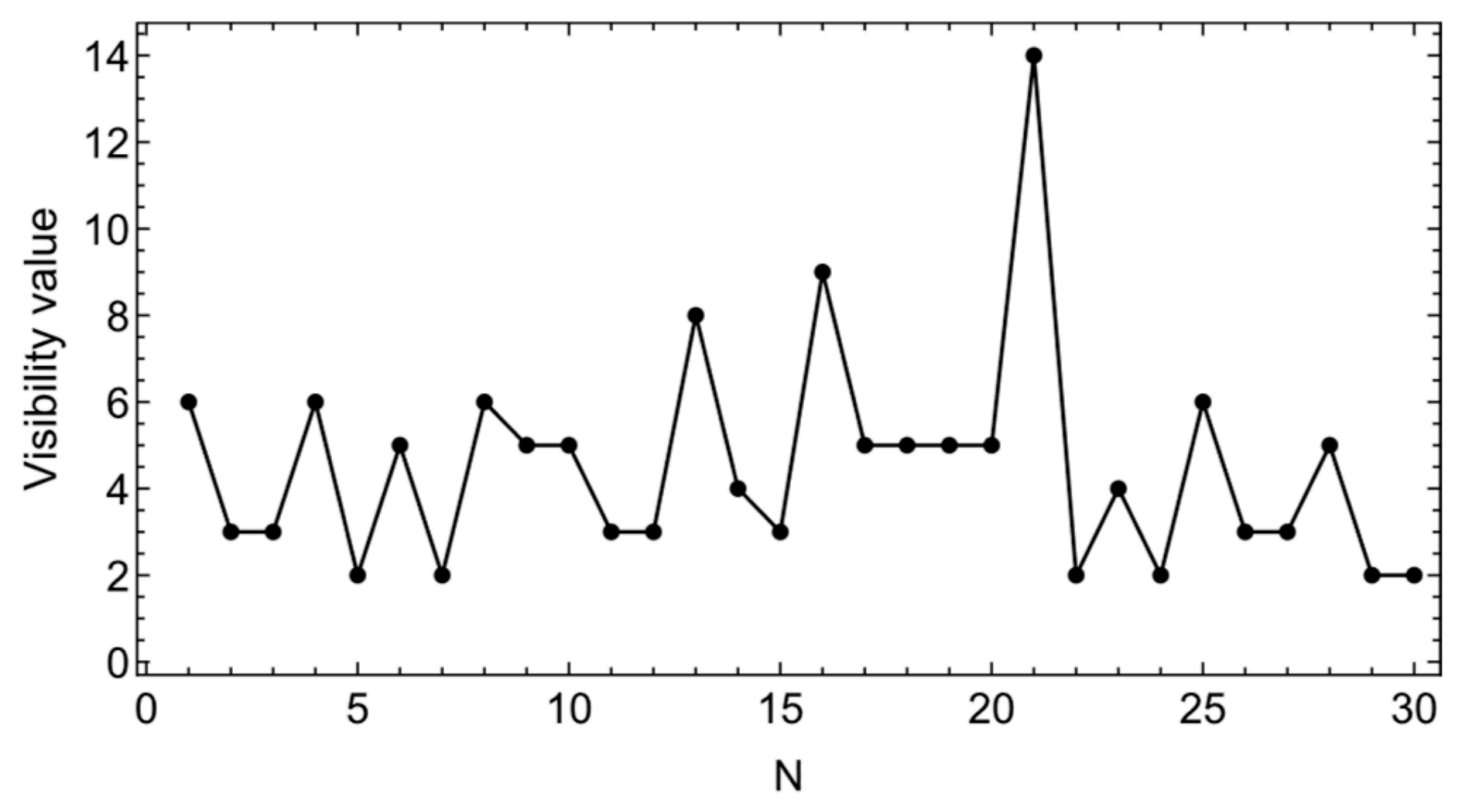
Visibility values for all points in the data series shown in Figure 2.

In the right panel of Figure 2, an example of visibility for two points (N=6 and N=16; marked as blue dots) is shown. Their corresponding visibility values are 5 and 9, respectively, and are visualized with red lines. The visibility values for all points in the example data series (Figure 2, left panel) are shown in Figure 3.

This data conversion provides insight into the hidden dynamic behaviour and interactions within the system under study, revealing correlations and dependencies that are not often visible at the level of input data. Such a data transformation was applied to all spectra analyzed in this study. Chalazion spectra were divided into two groups: without lipid disorders (A) and with lipid disorders (B). Serum spectra were compared across three groups: healthy control (K), patients with chalazions without lipid disorders (A), patients with chalazions with lipid disorders (B). Then we calculate the appropriate differences: A–B for chalazions, and A–B, A–K, B–K for serum spectra. For each comparison, visibility values were first calculated separately for each individual spectrum; group-level mean visibility profiles were then obtained, and the A–B, A–K, and B–K curves represent point-by-point differences between these group-averaged visibility profiles rather than differences computed from a single averaged Raman spectrum. The VG transformation differs from direct spectral-intensity analysis. The visibility value assigned to a Raman-shift position depends on its geometric relation to other points in the ordered spectrum and not only on its absolute intensity. The resulting VG profile therefore describes the local geometric organization of the spectrum while retaining the Raman-shift position of each point [26,27]. Local VG extrema were identified descriptively, without a statistical significance threshold or effect-size criterion.

An additional property of the natural visibility-graph transformation is its invariance under vertical translation, positive rescaling of signal amplitude, and addition or removal of a linear trend [26]. Consequently, constant offsets, overall positive intensity scaling, and linear baseline components do not alter the visibility relations between spectral points. This property does not imply invariance to arbitrary nonlinear backgrounds or to genuine substrate-related spectral features.

The VG approach was used as a complementary representation rather than as a replacement for established signal-analysis methods, including wavelet-based multiresolution analysis, entropy-based complexity measures, and two-dimensional correlation spectroscopy [28,29,30]. These methods describe different aspects of spectral data. Wavelet analysis examines structures at different scales, entropy measures quantify signal complexity, and correlation spectroscopy examines coordinated changes between spectral variables.

For the VG analysis, visibility values were calculated separately for each recorded spectrum and were then summarized within the study groups. Because several spectra could originate from the same biological sample, the resulting group profiles represent exploratory measurement-level summaries. They should not be interpreted as independent patient-level estimates or as evidence of diagnostic classification.

#### 2.3.4. Statistical Analysis

A statistical analysis was performed using GraphPad Prism (version 6, GraphPad Software, San Diego, CA, USA). The data distribution was assessed for normality. As the data did not meet the assumptions of normal distribution and the sample sizes were limited, non-parametric statistical tests were applied. Comparisons among three independent groups, control subjects (K), patients with chalazion without lipid disorders (A) and patients with chalazion and lipid disorders (B), were performed using the Kruskal–Wallis test. When a statistically significant difference was detected, the Dunn’s multiple comparison test was applied as a post hoc analysis to identify the differences between the groups. All statistical tests were two-tailed and a *p*-value of <0.05 was considered statistically significant. Data are presented as mean ± standard deviation (SD). All calculations related to visibility graphs were performed using Wolfram Mathematica (version 14.0, Wolfram Research, Inc., Champaign, IL, USA). Because of the limited number of independent patients, no supervised classification model, sensitivity, specificity, accuracy, ROC/AUC analysis, or external validation was performed. Multiple spectra obtained from the same biological sample were treated as technical measurements and were not used as independent diagnostic cases. Dividing these spectra between training and test sets could introduce patient-level data leakage and produce overly optimistic results. PCA and VG were therefore used only for exploratory visualization and identification of candidate spectral features.

## 3. Results and Discussion

### 3.1. Raman Spectra of Chalazion Secretion

**Figure 4:**
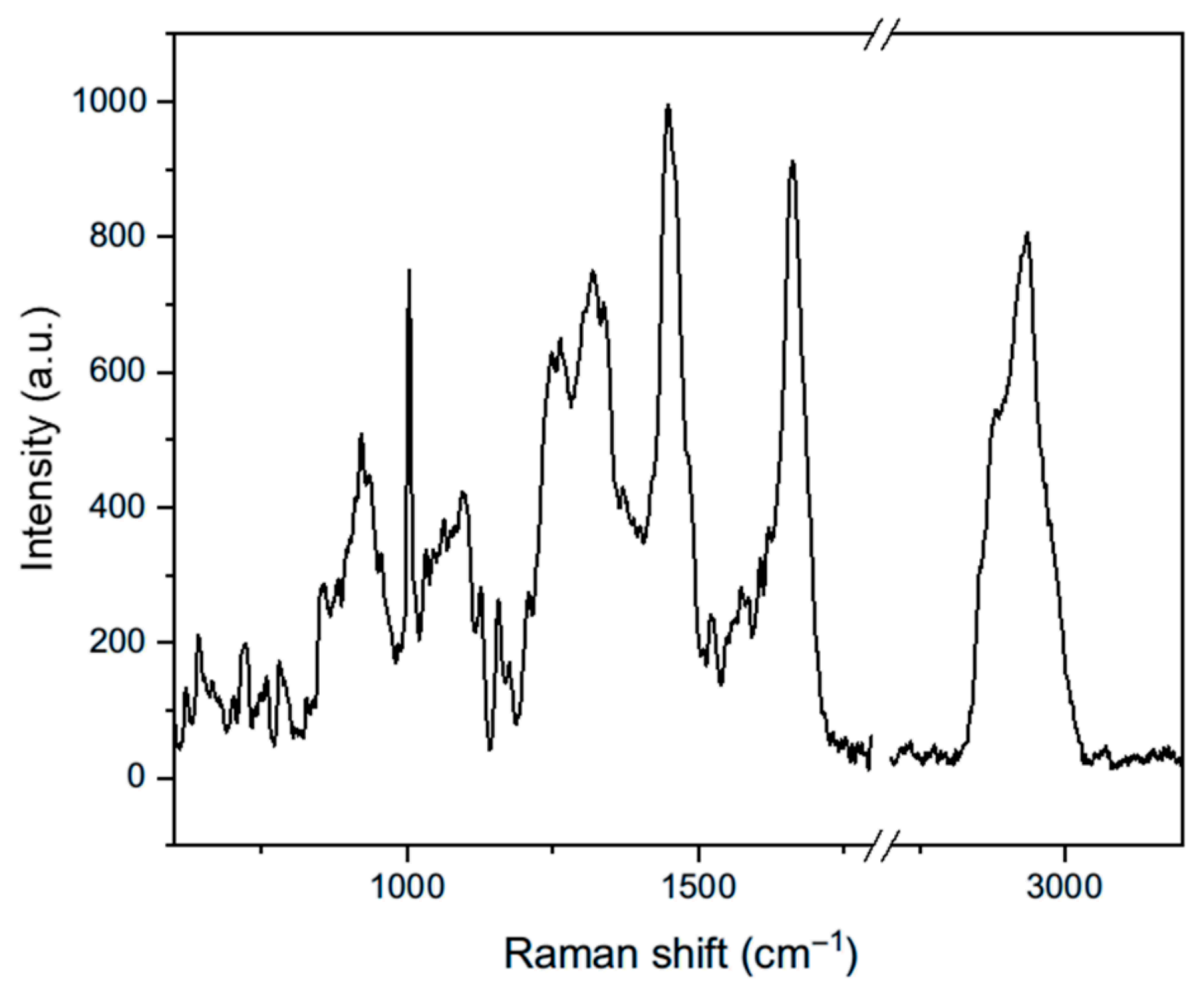
Raman spectra of chalazion secretion recorded in the spectral range of 600–3200 cm^−1^.

The first aim of the study was to characterize the Raman spectra of chalazion secretions produced by meibomian glands (MGs). The representative Raman spectrum is presented in Figure 4. Meibum is a lipid secretion composed of cholesteryl esters (CEs), cholesterol (Chl), triacylglycerols (TAG), waxes and other lipid types [8]. The observed spectra contained bands characteristic of the mentioned lipid structures, but the spectral features associated with different biological molecules were also observed. The signals related to proteins and nucleic acids were numerous, but their intensity remained relatively low. These signals may indicate the presence of cells in the secretion, including inflammatory cells—primarily neutrophils, lymphocytes, and macrophages—which participate in the body’s inflammatory response, as well as dead cells, cellular debris resulting from cell damage or death due to chronic inflammation. Slight variations in the spectra were observed depending on the measurement site for a given sample. This is due to the fact that the area of data acquisition was about 1 µm, and the spectrum can change when measurements encounter cells or lipid bodies. The detailed characterization of Raman bands observed in chalazion secretion spectra, based on reference assignments [23,24,25,31,32], is provided in Supplementary Table S1.

The obtained Raman spectra of chalazion secretions were compared between two groups: patients with and without diagnosis of lipid disorders.

**Figure 5:**
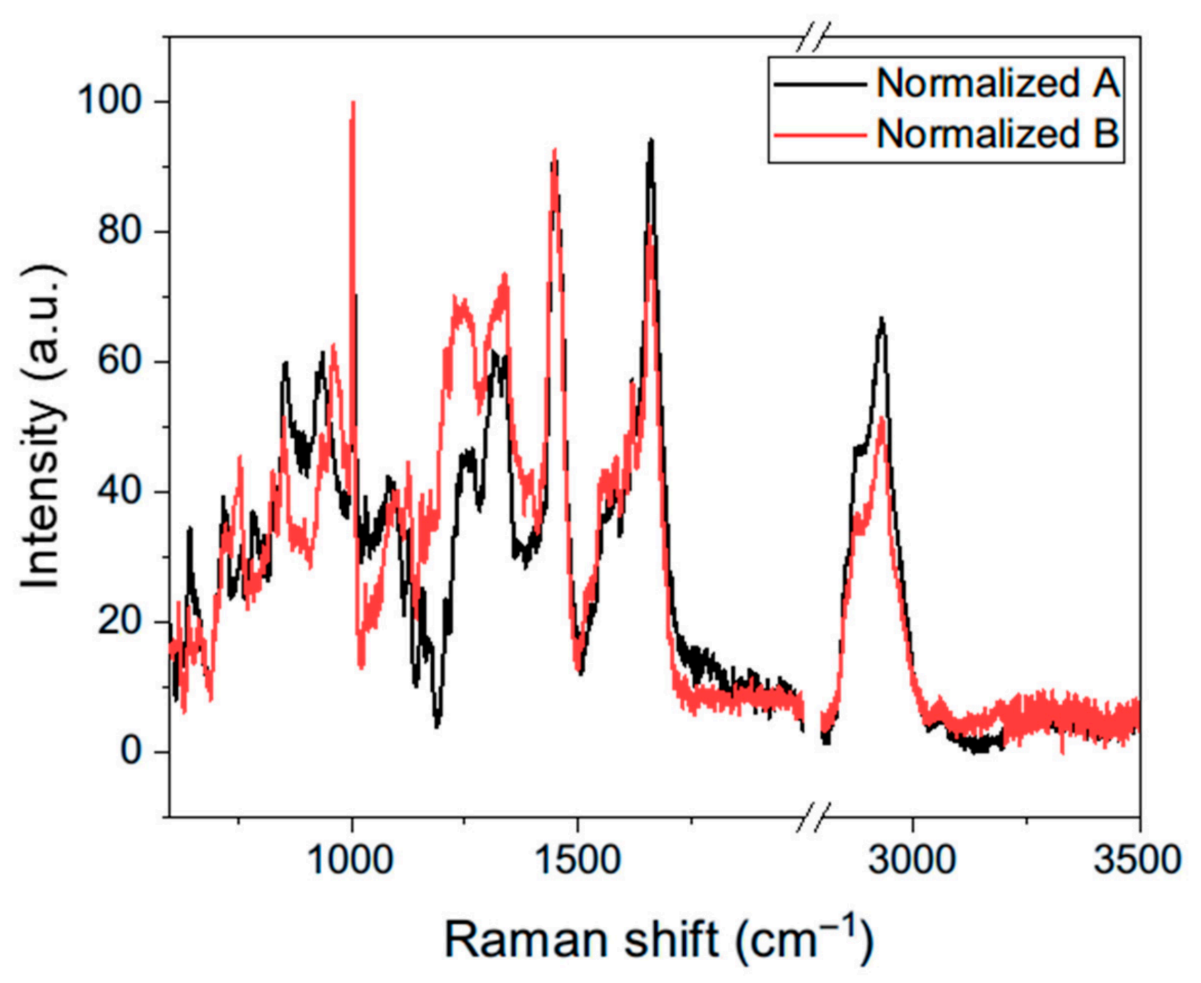
Raman spectra of chalazion secretions from patients without lipid disorders (A) and with lipid disorders (B).

**Figure 6:**
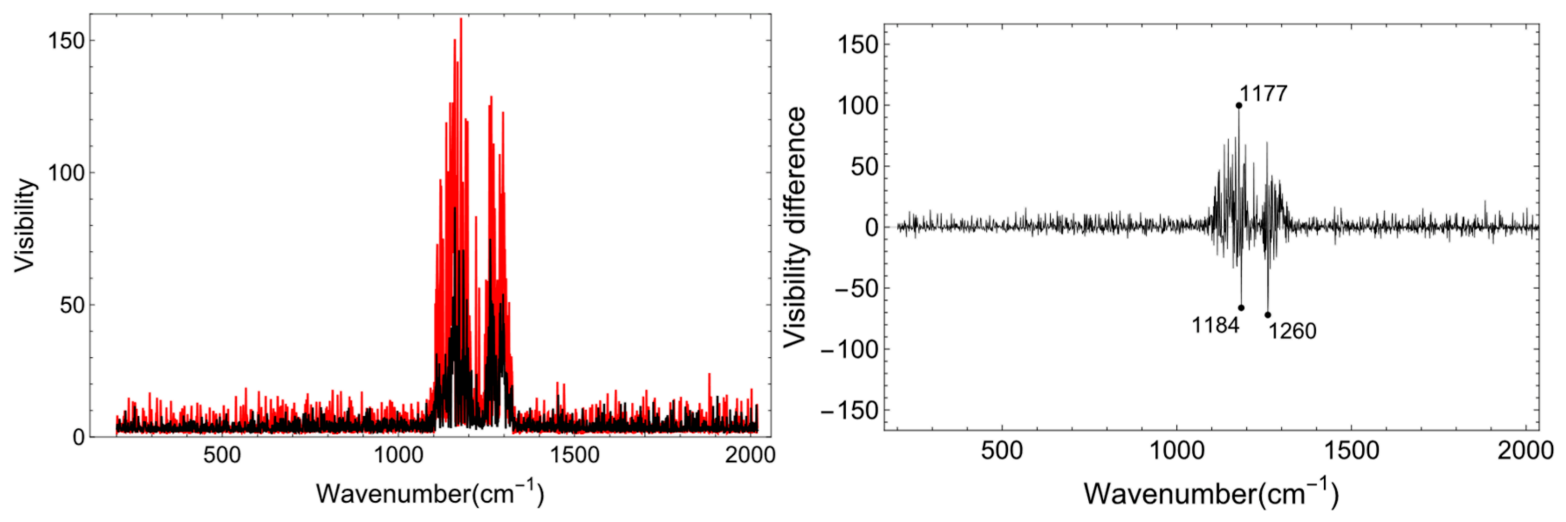
The average visibility (**left**) and the A–B visibility difference (**right**) of Raman spectra obtained from chalazion secretions in the 200–2000 cm^−1^ range. Localized extrema are observed near 1177, 1184, and 1260 cm^−1^.

The spectra are presented in Figure 5. Differences between individual spectra reflect inter-individual and local measurement variability. The full 200–2000 cm^−1^ range was first examined using the visibility-graph representation. The A–B VG difference profile is not distributed uniformly over the full range; the clear localized extrema occur within 1100–1300 cm^−1^, near 1177, 1184, and 1260 cm^−1^ (Figure 6). These positions overlap Raman regions with contributions from lipid-related C–C and =CH vibrations and protein-related signals, including amide III [23,24,25]. Because these bands overlap, the VG extrema are treated as candidate spectral positions rather than assignments to individual molecular compounds.

**Figure 7:**
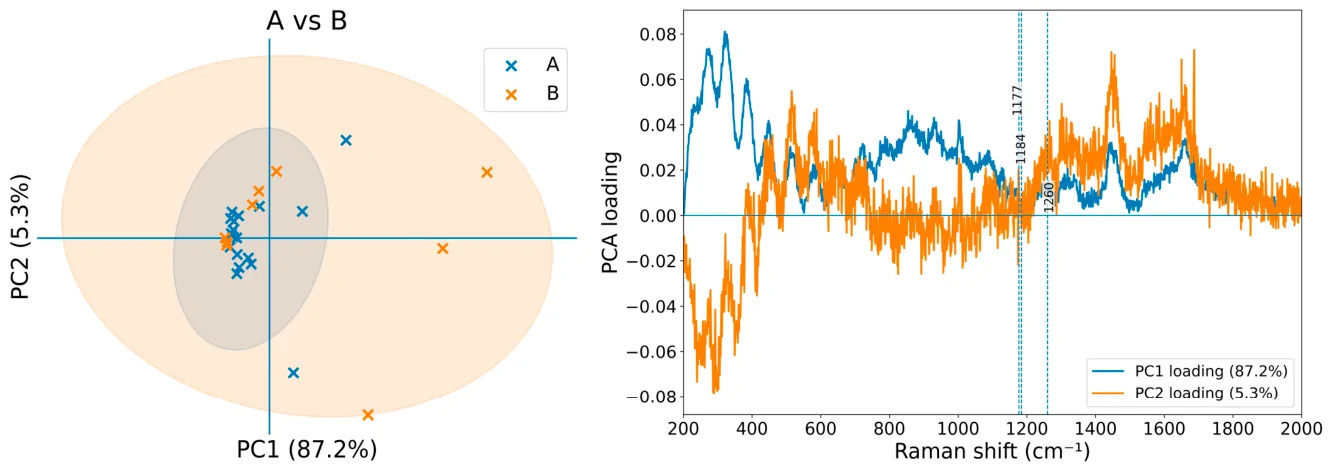
Full-range PCA of chalazion secretion (A vs. B), calculated over 200–2000 cm^−1^. **Left**: PC1–PC2 score plot with 95% covariance ellipses for the technical spectra. **Right**: corresponding PC1 and PC2 loading profiles; dashed vertical lines indicate the VG positions near 1177, 1184, and 1260 cm^−1^.

Full-range PCA provides a different view of the same A–B data (Figure 7). PC1 explains 87.2% of the total variance and PC2 5.3%. Despite the high contribution of PC1, the A and B covariance ellipses overlap extensively, and the broad B ellipse reflects substantial measurement-level variability. Thus, the dominant full-spectrum variance does not produce a clear A–B separation. The loading profiles contain contributions across several parts of the spectrum. The low-wavenumber region, particularly below approximately 600 cm^−1^, was retained in the PCA calculation but was not used for direct biochemical assignment because pronounced variability in this region may include substrate-related, measurement-related, and residual-background contributions. Small oscillatory structures were likewise not assigned to a unique origin, as measurement effects and physicochemical reorganization of the dried biological material cannot be separated with the present data. Above this low-wavenumber region, several loading features are chemically interpretable. In the 1400–1800 cm^−1^ range, structures around 1446 cm^−1^ overlap CH_2_/CH_3_ and C–H deformation bands associated with lipids and proteins, whereas features around 1650–1661 cm^−1^ overlap C=C and amide I contributions. These features show that full-range PCA contains meaningful spectral information, but its dominant covariance structure does not isolate the same localized 1100–1300 cm^−1^ pattern identified by VG.

**Figure 8:**
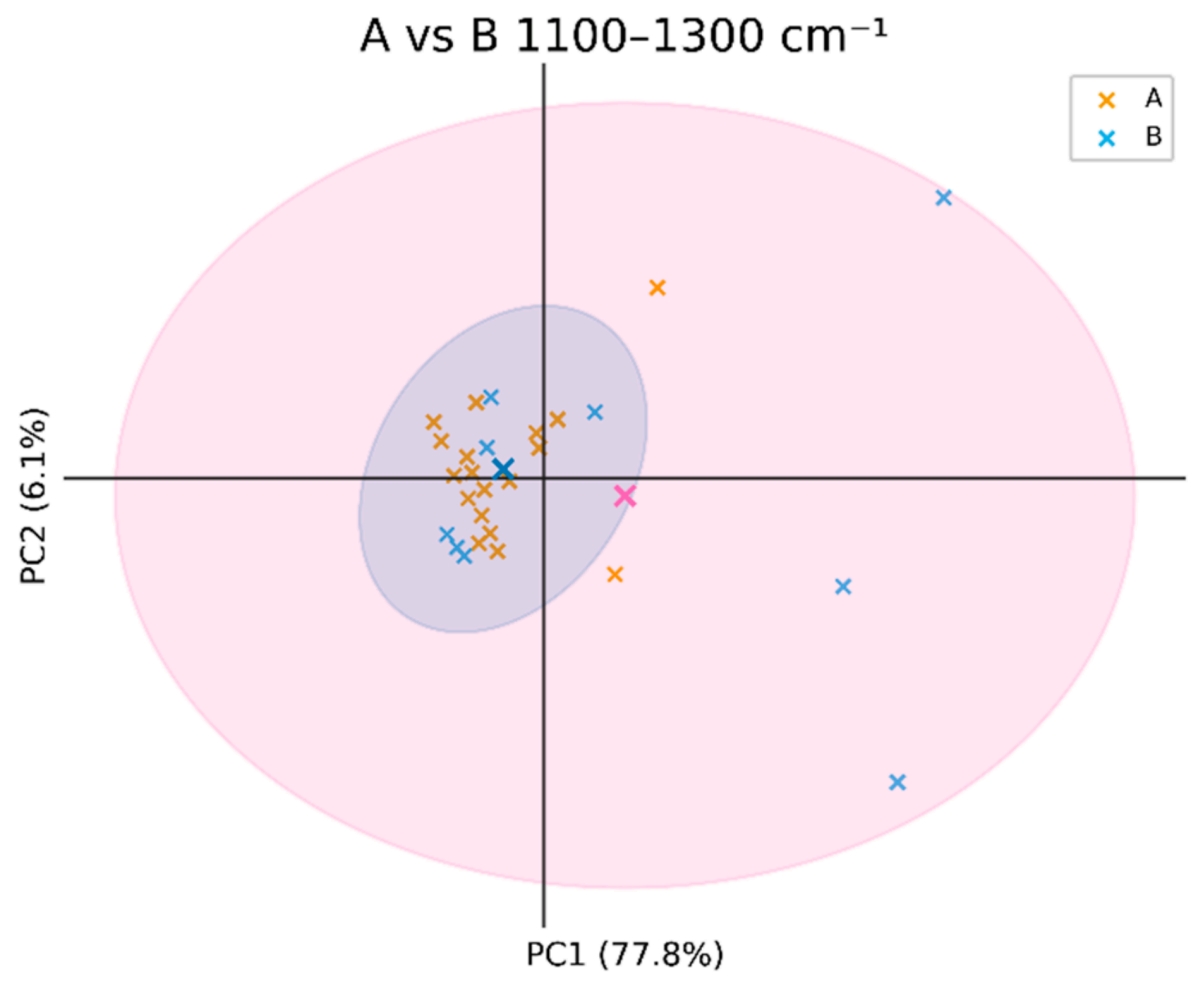
PCA of Raman spectra obtained from chalazion secretions of patients without lipid disorders (A) and with lipid disorders (B) in the 1100–1300 cm^−1^ range. The ellipses describe the distribution of technical spectra at the measurement level. Distant observations were retained because no objective exclusion criterion was met.

PCA was then examined in the 1100–1300 cm^−1^ range indicated by VG. For chalazion secretion A vs. B, PC1 explains 77.8% of the variance and PC2 6.1% (Figure 8). The group B ellipse is extensive and partially encloses the ellipse of group A, and two distant B observations substantially increase its size. The covariance ellipses therefore show substantial overlap and the centroid displacement along PC1 is limited. These distributions describe technical spectra at the measurement level and do not constitute patient-level classification. A sensitivity analysis excluding the two distant observations changed the explained variance to PC1 = 67.4% and PC2 = 8.3% and reduced the apparent centroid displacement. No objective acquisition or preprocessing error or predefined exclusion criterion was identified, so these observations were retained.

Within the 1100–1300 cm^−1^ PCA model, the combined PC1–PC2 loading magnitudes at approximately 1177, 1184, and 1260 cm^−1^ were 0.074, 0.055, and 0.121, corresponding to approximately the 32nd, 16th, and 73rd percentiles among the 183 variables. The VG extremum near 1184 cm^−1^ is therefore weak in the first two PCA loadings, whereas the position near 1260 cm^−1^ is represented by both methods. The strongest PCA loading features occur at other positions, including approximately 1296, 1271, and 1132 cm^−1^. This illustrates the different information emphasized by the two methods: PCA ranks variables according to their contribution to covariance, whereas VG directly preserves the Raman-shift locations at which the group-averaged geometric profiles differ.

#### Raman Spectra of Serum

The next step was to consider using Raman spectroscopy to analyze patient serum. Serum is routinely used in laboratory diagnostics and can be analyzed alongside standard biochemical tests. Raman spectroscopy has been successfully used to study serum as described in the literature (e.g., [33,34,35]). In the studies presented here, Raman spectroscopy was used to analyze the serum of people divided into three groups: patients with chalazion (group A), patients with chalazion and lipid disorders (group B), and a control group of healthy men (group K).

**Figure 9:**
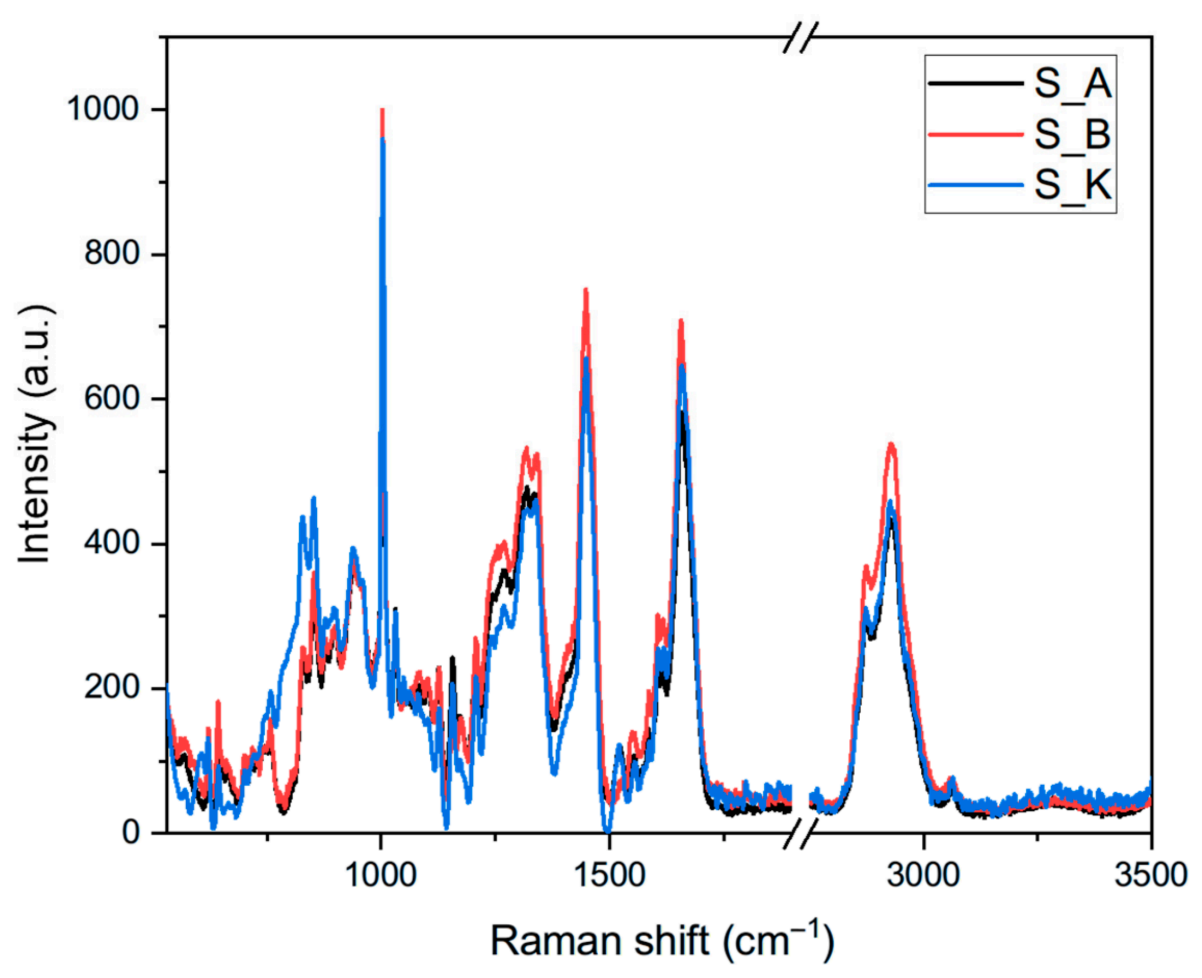
Raman spectra of serum: control (K), patients with chalazion (A), patients with chalazion and with lipid disorders (B).

Figure 9 presents the averaged Raman spectra of serum for groups A, B, and K. The Raman spectral bands can be identified as can the biological molecules associated with them. The serum spectrum shows numerous bands that can be attributed to proteins. Examples include 1661 cm^−1^ (amide I), 1555 cm^−1^ (amide II), 1271 cm^−1^ (amide III), 1127 cm^−1^ and 758 cm^−1^. Some of these bands are attributed to the vibrations of the specific amino acid structures, such as 1606 cm^−1^ (Tyr, Phe, Cys), 1584 cm^−1^ (Phe, Arg, Ade), 1207 cm^−1^ (Trp, Tyr, Phe), 1002 cm^−1^ (Phe), 854 cm^−1^ (Tyr, Pro). C-H vibrations, which are mainly associated with lipids, produce signals at 2800–3100 cm^−1^ (stretching vibrations) and 700–1500 cm^−1^ (deformation vibrations). Lipid bands are also identified in other types of vibrations: C-C, C=C, and skeletal vibrations. The detailed characterization of Raman bands identified in the serum spectra, based on reference assignments [25,32,33,34,35,36,37], is provided in Supplementary Table S2.

**Figure 10:**
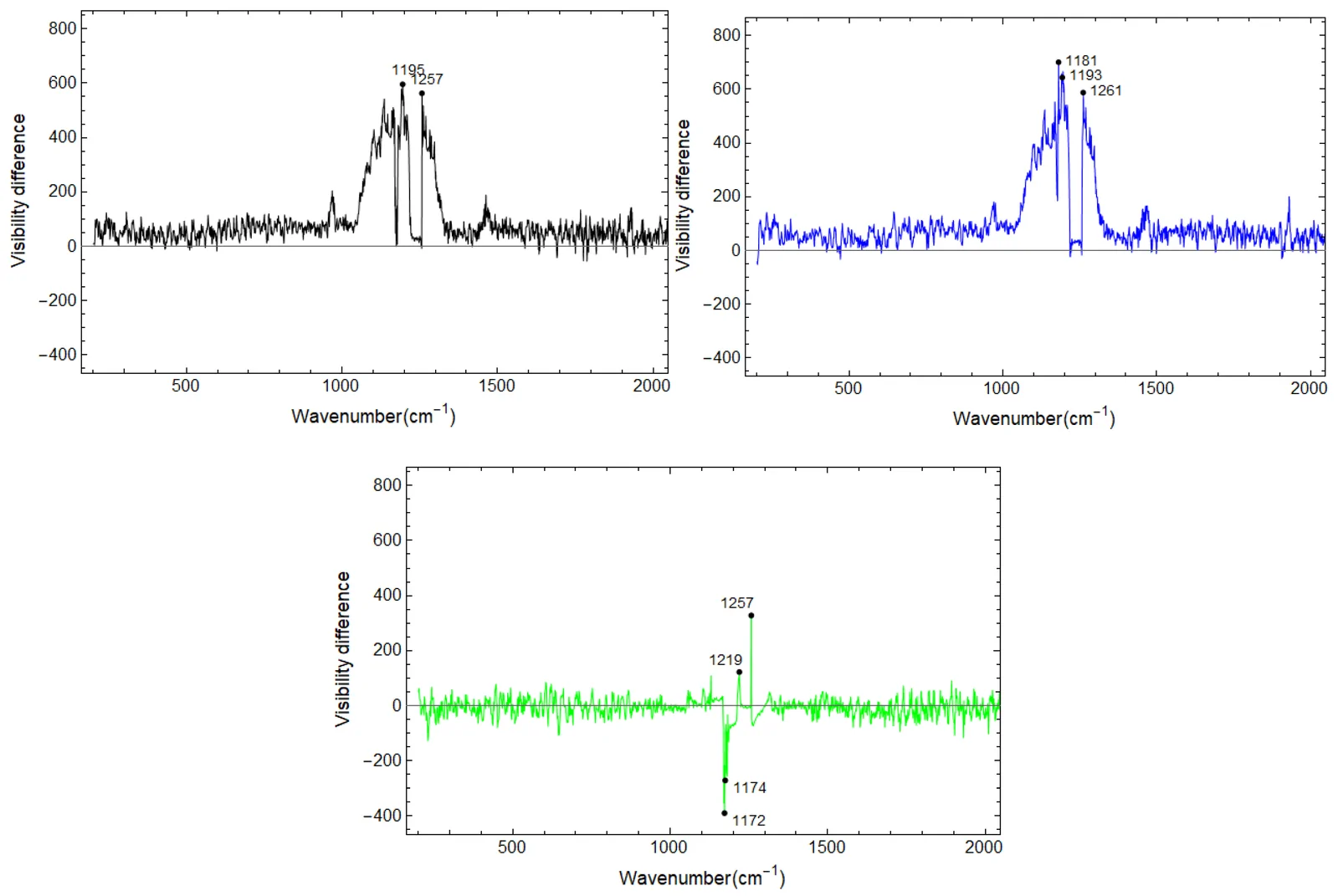
Visibility differences in Raman spectra obtained from serum over 200–2000 cm^−1^: group A vs. K (black), group B vs. K (blue), and group A vs. B (green).

The full 200–2000 cm^−1^ serum range was first examined using VG for all three pairwise comparisons (Figure 10). The A–K and B–K visibility-difference profiles show similar overall behaviour, whereas A–B shows a distinct localized pattern. For A–B, local extrema occur near 1172, 1174, 1219, and 1257 cm^−1^. Localized extrema for A–K and B–K also occur within the 1100–1300 cm^−1^ range. These positions overlap Raman regions with lipid- and protein-related contributions and are treated as candidate spectral positions rather than compound-specific markers.

**Figure 11:**
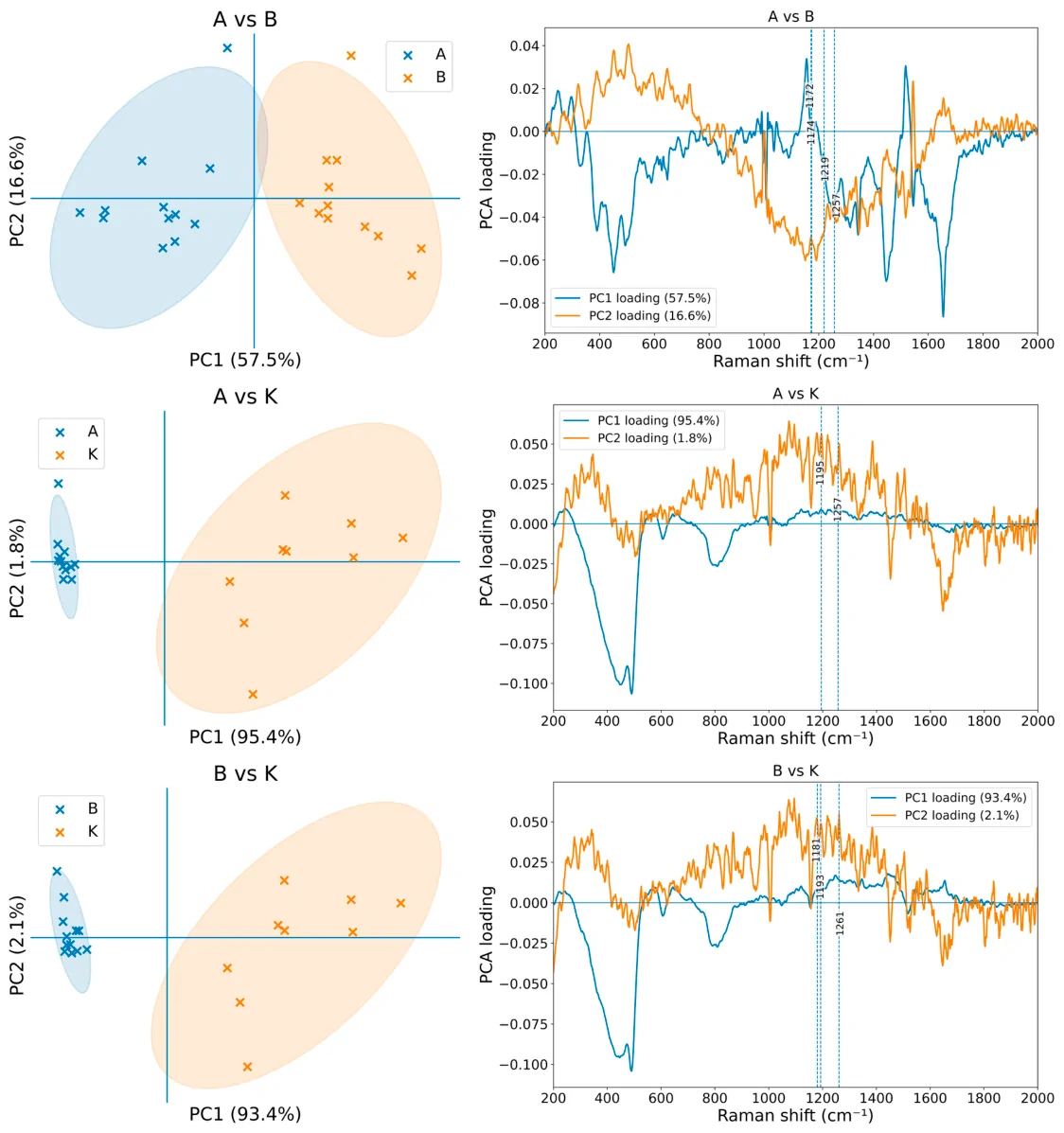
Full-range PCA of serum, calculated over 200–2000 cm^−1^. Left column: PC1–PC2 score plots with 95% covariance ellipses for A vs. B, A vs. K, and B vs. K. Right column: corresponding PC1 and PC2 loading profiles; dashed vertical lines indicate the localized positions identified by VG for each comparison.

The full-range serum PCA models show different group-distribution patterns and loading structures (Figure 11). For A–B, PC1 and PC2 explain 57.5% and 16.6% of the variance, respectively. The groups are separated mainly along PC1, although the covariance ellipses retain partial overlap. In contrast, A–K and B–K show very strong separation and are dominated by PC1, which explains 95.4% and 93.4% of the variance, while PC2 explains only 1.8% and 2.1%. The pronounced low-wavenumber loading structure in the comparisons with K, especially below approximately 600 cm^−1^, was retained in the PCA models but was not assigned directly to biological constituents. This region may contain contributions from the quartz substrate, measurement-related effects, and residual background variability; therefore, its molecular interpretation is uncertain. In the higher-wavenumber part of the spectrum, several loading features are physically and chemically interpretable. In serum A–B, strong PC1 contributions occur near 1447 cm^−1^ and 1655–1659 cm^−1^. The 1446–1447 cm^−1^ region overlaps CH_2_/CH_3_ and C–H deformation bands associated with triacylglycerols, fatty acids, cholesterol, cholesterol esters, and proteins, whereas 1655–1659 cm^−1^ overlaps C=C and amide I contributions from lipids and proteins. Thus, full-range PCA contains meaningful biochemical information; however, these contributions are distributed across several spectral regions and do not isolate the same localized 1100–1300 cm^−1^ pattern obtained with VG.

**Figure 12:**
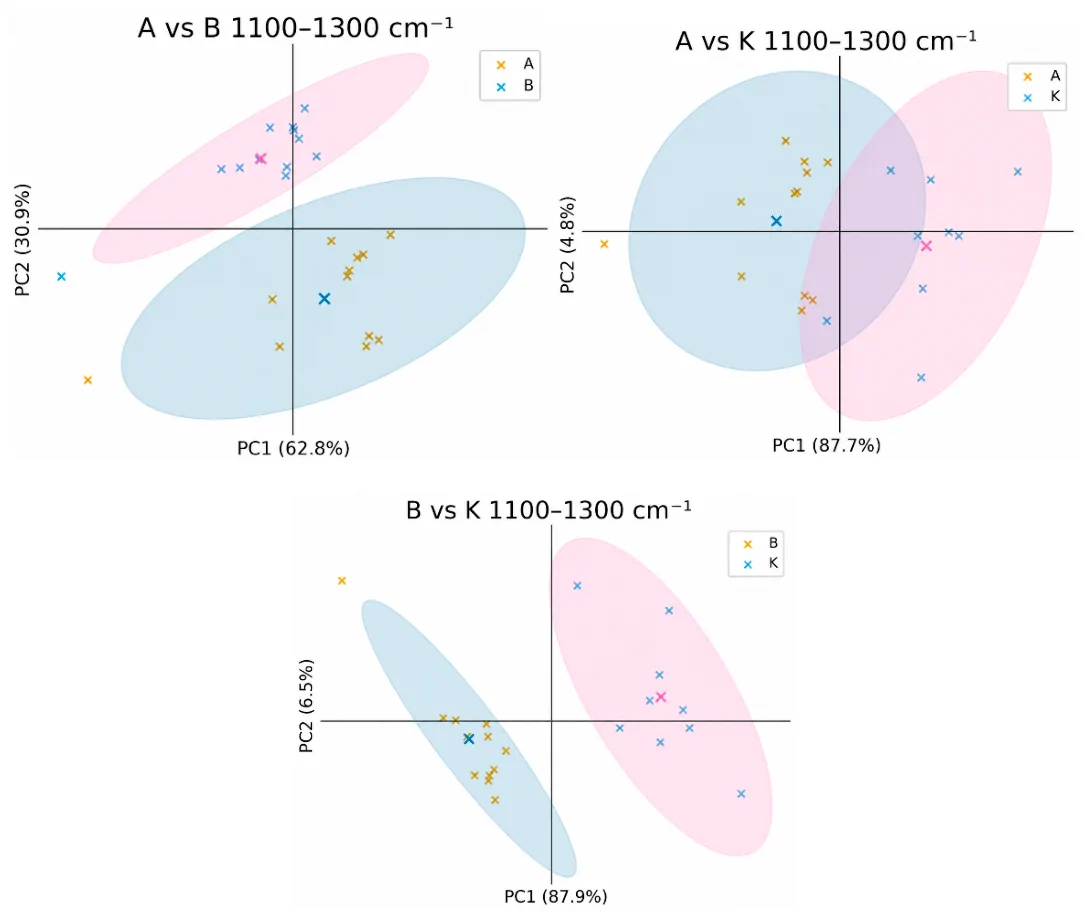
PCA of Raman spectra obtained from serum in the 1100–1300 cm^−1^ range: patients with chalazion without lipid disorders (A) versus patients with chalazion and lipid disorders (B), A versus control (K), and B versus control (K). The ellipses describe the distribution of technical spectra at the measurement level.

PCA was then examined in the 1100–1300 cm^−1^ range indicated by VG (Figure 12). For serum A–B, PC1 and PC2 explain 62.8% and 30.9% of the variance. The covariance ellipses are separated and oriented differently, so both leading components contribute to the observed group structure. As in the chalazion-secretion analysis, these ellipses describe distributions of technical spectra and should be interpreted descriptively rather than as validated patient-level discrimination.

For A–K and B–K in the same 1100–1300 cm^−1^ range, PC1 explains 87.7% and 87.9% of the variance, respectively, while PC2 explains 4.8% and 6.5%. The separation from group K is more visible for B–K, whereas A–K shows greater overlap. These results indicate that the PCA geometry within the VG-indicated range differs between the pairwise comparisons, while the VG profiles retain direct information on the Raman-shift positions of the localized differences.

For serum A–B in the 1100–1300 cm^−1^ PCA model, the combined loading magnitudes at the VG positions near 1172, 1174, 1219, and 1257 cm^−1^ were 0.08, 0.08, 0.077, and 0.124, corresponding to approximately the 19th, 20th, 12th, and 82nd percentiles. Thus, the VG changes near 1172, 1174, and 1219 cm^−1^ are not prominent in PC1 or PC2, whereas the feature near 1257 cm^−1^ is indicated by both methods. The strongest combined loading occurs at 1155.9 cm^−1^ for A–B, 1232.5 cm^−1^ for A–K, and 1150.4 cm^−1^ for B–K. The absence of one dominant loading position common to the serum models reflects the pair-dependent covariance structure of PCA, whereas VG directly compares the group-averaged local spectral geometry.

Similar conclusions are drawn from the analysis of the intensities of selected bands (Figure 9). Higher intensities of the bands in the 1100–1300 cm^−1^ region, as well as the bands at 1316, 1447, 2931, 2875 cm^−1^ associated with the C-H bond vibrations originating mainly from lipids, were recorded for the samples in group B. This finding is associated with increased levels of lipid content in the blood (including triglycerides and cholesterol) of patients with lipid disorders. The band 1659 cm^−1^, which arises from vibrations from lipids (including cholesterol) and proteins (amide I: C=O and N-H bonds [25]), was also analyzed in detail. Additionally, the intensity ratio of I1447/I1659 was evaluated as a candidate indicator of disorders, including cancer, as reported in the literature [32,33,34,35]. For the control group the I1447/I1659 ratio was 1.04 ± 0.05, whereas for patients with chalazion the I1447/I1659 ratio was 0.93 ± 0.05.

However, the spectral differences associated with the presence of chalazion as observed in the examined groups—four patients in group A, four patients in group B and three patients in the control group—are not statistically significant. The results may indicate a relationship, but they do not provide conclusive evidence. It is possible that other as yet unexplored diseases affect the obtained results, or the research group should be significantly enlarged to improve the statistics.

The apparently clearer separation observed in serum than in chalazion secretion may reflect the fact that serum more directly represents systemic lipid dysregulation, whereas chalazion secretion is a local lesion-derived material influenced by inflammation stage, lesion duration, cellular debris, local tissue response, and measurement-site heterogeneity. Thus, serum may provide a more homogeneous biochemical matrix, while chalazion secretion may contain a stronger local and spatially heterogeneous component. This interpretation remains preliminary and requires verification in a larger cohort.

Taken together, the full-range analyses show that PCA and VG emphasize different aspects of the Raman data. PCA captures dominant covariance and, in several comparisons, shows both group separation and chemically meaningful loading features. These contributions are distributed across multiple spectral regions. VG, applied over the same broad range, localizes the pairwise differences within 1100–1300 cm^−1^ and retains their Raman-shift positions. The natural visibility graph is also invariant to vertical translation, positive amplitude rescaling, and addition or removal of a linear trend [26], whereas PCA can reflect broad intensity covariance associated with residual baseline variability. This mathematical property can reduce the influence of affine baseline components in VG, but it does not make VG insensitive to arbitrary nonlinear backgrounds or genuine substrate features.

PCA and VG therefore provide complementary views of the data. The distinction in the present study concerns spectral localization: full-range PCA summarizes the dominant global covariance structure, whereas VG identifies a localized set of pairwise differences within 1100–1300 cm^−1^. PCA in this range provides the corresponding multivariate representation for direct comparison with the VG-indicated spectral interval.

### 3.2. Study Limitations

This study has several important limitations. First, the number of patients was small, particularly in the serum control group. Second, the age range was broad and the groups were not fully balanced in terms of sex distribution. Third, medication use, metabolic status, diet, and the inflammatory stage or duration of chalazion may have influenced both lipid profiles and Raman spectra. Fourth, the absence of an independent validation cohort prevents assessment of generalizability. Therefore, the present findings should be interpreted as exploratory and hypothesis-generating.

The limited and unbalanced cohort composition precluded adjustment for age, sex, medication use, metabolic status, lesion duration, and other potential confounding variables. In particular, the three-subject, all-male serum control group cannot establish a representative healthy baseline. Moreover, the absence of a healthy secretion control means that the secretion analysis cannot distinguish changes associated with chalazion itself from changes associated with lipid-disorder status. The observed patterns should therefore be regarded as preliminary, descriptive, and hypothesis-generating.

Multiple spectra recorded from the same biological sample were used to describe local spectral heterogeneity. These spectra were technical measurements and were not independent at the patient level. Their inclusion may affect the apparent shape and spread of the PCA distributions. Therefore, PCA and VG results were interpreted only as exploratory measurement-level patterns. Patient-level averaging and formal validation should be performed in larger cohorts.

A meaningful confirmatory extension of this work would require a prospectively designed cohort with a predefined sample-size calculation, balanced age and sex distributions, and patient-level statistical analysis. Adding a small number of additional samples to the present dataset would not resolve these design limitations.

Both VG and PCA were evaluated over the full 200–2000 cm^−1^ range, and PCA was additionally examined within 1100–1300 cm^−1^ because VG localized the clearest pairwise differences in this interval. The full-range PCA results include contributions from low-wavenumber regions that cannot be assigned unambiguously to the biological samples because substrate-related, measurement-related, and residual-background effects may contribute. The present study therefore does not use the pronounced features below approximately 600 cm^−1^ for molecular interpretation. In addition, the PCA distribution for chalazion secretion remains sensitive to two distant spectra from group B; their removal was not justified by an objective measurement-quality criterion. These considerations, together with the limited number of independent patients, restrict the strength of mechanistic or diagnostic interpretation.

The present dataset is preliminary and exploratory in nature and, therefore, does not support the reliable estimation of diagnostic performance. Sensitivity, specificity, accuracy, and ROC/AUC values would be unstable because of the small number of independent subjects. Treating repeated spectra from the same sample as independent diagnostic cases would also introduce pseudoreplication. Diagnostic validation will require a larger prospectively designed cohort, patient-level analysis, and an independent test set.

## 4. Conclusions

The present exploratory study suggests an association between systemic lipid alterations and biochemical differences detected in chalazion secretions and patient serum. Serum analysis revealed higher total cholesterol, triglyceride and LDL levels in patients with chalazion and lipid disorders (group B), with only minor variations in HDL levels between groups. These biochemical findings are consistent with Raman spectroscopic observations of lipid-related spectral differences primarily within the 1100–1300 cm^−1^ region. This concordance supports a possible relationship between systemic dyslipidemia and the biochemical composition of chalazion-related material, but it should not be interpreted as diagnostic validation. The VG transformation provided a Raman-shift-resolved representation of geometric changes within the spectra. The identified VG extrema should be treated as candidate spectral positions and not as statistically validated biomarkers. Because the corresponding Raman bands contain overlapping lipid- and protein-related contributions, the present data do not support their assignment to individual molecular compounds. The numerical comparison with the PCA loadings showed that some VG positions were also associated with relatively large PC1 or PC2 contributions, while other strong VG extrema were weak in both loading profiles. This indicates that VG can identify localized group-related spectral changes that are not among the dominant sources of total variance captured by PCA. The VG-derived spectral positions may provide candidate features for future diagnostic models, but the present results do not constitute diagnostic validation. More broadly, this graph-based approach may be relevant for studies in which complex biomedical signals are transformed into network representations to support the extraction and interpretation of localized information. In this context, visibility-graph analysis may also provide a useful basis for future extensions involving entropy-based or other information-theoretic measures of spectral complexity. Further studies involving larger and better balanced sample sets, subject-level statistical analysis, ROC/AUC assessment, sensitivity/specificity estimation, cross-validation, permutation testing, and independent external validation are required before any diagnostic application can be proposed. Full-range PCA captured global covariance structure and included chemically interpretable spectral contributions, including features around 1446–1447 and 1655–1659 cm^−1^. However, the dominant loading structure was distributed across several parts of the spectrum and did not isolate the same localized 1100–1300 cm^−1^ pattern identified by VG. VG therefore provided a complementary Raman-shift-resolved localization of the pairwise group differences within this range. The low-wavenumber PCA features below approximately 600 cm^−1^ were retained in the calculations but were not used for biochemical assignment because their origin may include substrate-related, measurement-related, and residual-background contributions.

## Figures and Tables

**Table 1:** Patient details.

	Chalazion Secretion	Serum
Gender male/female	8/8	4/7
Age range (median) [years]	15–69 (44)	14–72 (41)
Patients without lipid disorders	7	4
Patients with lipid disorders	9	4
Serum control samples	-	3

## Data Availability

The datasets generated and analyzed during this study are available from the corresponding author upon reasonable request due to ethical and privacy restrictions.

## Supplementary Materials

The following supporting information can be downloaded at: https://www.mdpi.com/article/10.3390/e28091011/s1, Table S1: Raman band assignments of spectra obtained from chalazion secretion [21,22,23,24,25]; Table S2: Raman band assignments of serum [24,25,26,27,28,29,30].

## References

[1] Chhadva P., Goldhardt R., Galor A. (2017). Meibomian gland disease. Ophthalmology.

[2] Jordan G.A., Beier K. (2023). Chalazion.

[3] Evans J., Vo K.B.H., Schmitt M. (2022). Chalazion: Racial risk factors for formation, recurrence, and surgical intervention. Can. J. Ophthalmol..

[4] Kim E.S., Afshin E.E., Elahi E. (2023). The lowly chalazion. Surv. Ophthalmol..

[5] Şen F., Zengin N. (2020). Meibomian Gland Dysfunction and Dyslipidemia. Harran Üniversitesi Tıp Fakültesi Derg..

[6] Arita R., Suehiro J., Haraguchi T., Shirakawa R., Tokoro H., Amano S. (2013). Objective image analysis of the meibomian gland area. Br. J. Ophthalmol..

[7] Wojtowicz J.C., Butovich I.A., McMahon A., Hogan R.N., Itani K.M., Mancini R., Molai M., Linsenbardt E. (2014). Time-dependent degenerative transformations in the lipidome of chalazia. Exp. Eye Res..

[8] Butovich I.A., Wilkerson A., Yuksel S. (2021). Differential effects of dietary cholesterol and triglycerides on the lipid homeostasis in Meibomian glands. J. Steroid. Biochem. Mol. Biol..

[9] Eberhardt K., Stiebing C., Matthäus C., Schmitt M., Popp J. (2015). Advantages and limitations of Raman spectroscopy for molecular diagnostics: An update. Expert Rev. Mol. Diagn..

[10] Thomas K.M., Ajithaprasad S.N.M., Pavithran M.S., Chidangil S., Lukose J. (2024). Raman spectroscopy assisted tear analysis: A label free, optical approach for noninvasive disease diagnostics. Exp. Eye Res..

[11] Butler H.J., Ashton L., Bird B., Cinque G., Curtis K., Dorney J., Esmonde-White K., Fullwood N.J., Gardner B., Martin-Hirsch P.L. (2016). Using Raman spectroscopy to characterize biological materials. Nat. Protoc..

[12] Bernstein P.S., Zhao D.Y., Sharifzadeh M., Ermakov I.V., Gellermann W. (2004). Resonance Raman measurement of macular carotenoids in the living human eye. Arch. Biochem. Biophys..

[13] Banbury C., Mason R., Styles I., Eisenstein N., Clancy M., Belli A., Logan A., Goldberg O.P. (2019). Development of the Self Optimising Kohonen Index Network (SKiNET) for Raman Spectroscopy Based Detection of Anatomical Eye Tissue. Sci. Rep..

[14] Katz A., Kruger E.F., Minko G., Liu C.H., Rosen R.B., Alfano R.R. (2003). Detection of glutamate in the eye by Raman spectroscopy. J. Biomed. Opt..

[15] Deng S., Dou W., Wu Y., Shao M., Ji C., Yu J., Zhang C., Xu S., Man B., Li Z. (2026). Dual-tunable SERS substrate: Synergistic enhancement via plasmonic resonance tailoring and pyroelectric field modulation in Ag/BTO hybrid arrays. Opt. Laser Technol..

[16] Xu S., Tian M., Li C., Liu G., Wang J., Yan T., Li Z. (2025). Using a crumpled graphene field-effect transistor for ultrasensitive SARS-CoV-2 detection via its papain-like protease. Chem. Eng. J..

[17] Tian M., Wei J., Lv E., Li C., Liu G., Sun Y., Yang W., Wang Q., Shen C., Zhang C. (2024). Drug evaluation platform based on non-destructive and real-time in situ organoid fate state monitoring by graphene field-effect transistor. Chem. Eng. J..

[18] Wu D., Ma Z., Yang X., Jiang G., Yan C., Ma L. Image Visibility Graph Analysis of the Eye Disease Images. Proceedings of the 16th International Congress on Image and Signal Processing, BioMedical Engineering and Informatics (CISP-BMEI).

[19] Mohammadpoory Z., Nasrolahzadeh M., Mahmoodian N., Haddadnia J. (2019). Automatic identification of diabetic retinopathy stages by using fundus images and visibility graph method. Measurement.

[20] Surmacki J.M., Woodhams B.J., Haslehurst A., Ponder B.A.J., Bohndiek S.E. (2018). Raman micro-spectroscopy for accurate identification of primary human bronchial epithelial cells. Sci. Rep..

[21] Fazio E., Corsaro C., Speciale A., Saija A., Salamone F.L., Mondello M., Sciaccotta R., Giordano L., Stagno F., Allegra A. (2026). Biochemical fingerprinting of dried blood serum from chronic lymphocytic leukemia patients by Raman spectroscopy: Towards prognostic classification. Spectrochim. Acta Part A Mol. Biomol. Spectrosc..

[22] Jolliffe I.T., Cadima J. (2016). Principal component analysis: A review and recent developments. Philos. Trans. R. Soc. A.

[23] Czamara K., Majzner K., Pacia M., Kaczor M., Barańska A., Kochan M. (2015). Raman spectroscopy of lipids: A review. J. Raman Spectrosc..

[24] Ryguła A., Majzner K., Marzec K.M., Kaczor A., Pilarczyk M., Barańska M. (2013). Raman spectroscopy of proteins: A review. J. Raman Spectrosc..

[25] Talari A.C.S., Movasaghi Z., Rehman S., Rehman I. (2015). Raman Spectroscopy of Biological Tissues. Appl. Spectrosc. Rev..

[26] Lacasa L., Luque B., Bllesteros F., Luque J., Nuno J.C. (2008). From time series to complex networks: The visibility graph. Proc. Natl. Acad. Sci. USA.

[27] Fano Y.D., Stowell D., Sandler M. Spectral Visibility Graphs: Application to Similarity of Harmonic Signals. Proceedings of the 27th European Signal Processing Conference (EUSIPCO).

[28] Mallat S.G. (1989). A theory for multiresolution signal decomposition: The wavelet representation. IEEE Trans. Pattern Anal. Mach. Intell..

[29] Bandt C., Pompe B. (2002). Permutation entropy: A natural complexity measure for time series. Phys. Rev. Lett..

[30] Noda I. (1993). Generalized two-dimensional correlation method applicable to infrared, Raman, and other types of spectroscopy. Appl. Spectrosc..

[31] Oshima Y., Sato H., Zaghloul A., Foulks G.N., Yappert M.C., Borchman D. (2009). Characterization of Human Meibum Lipid using Raman Spectroscopy. Curr. Eye Res..

[32] Gonzalez-Solis J.L., Martinez-Espinosa J.C., Torres-Gonzalez L.A., Aguilar-Lemarroy A., Jave-Suarez L.F., Palomares-Anda P. (2014). Cervical Cancer Detection Based on Serum Sample Raman Spectroscopy. Lasers Med. Sci..

[33] Huefner A., Kuan W.L., Mason S.L., Mahajan S., Barker R.A. (2020). Serum Raman spectroscopy as a diagnostic tool in patients with Huntington’s disease. Chem. Sci..

[34] Staritzbichler R., Hunold P., Estrela-Lopis I., Hildebrand P.W., Isermann B., Kaiser T. (2021). Raman spectroscopy on blood serum samples of patients with end-stage liver disease. PLoS ONE.

[35] Schie I., Huser T. (2013). Methods and applications of Raman microspectroscopy to single-cell analysis. Appl. Spectrosc..

[36] Movasaghi Z., Rehman S., Rehman I. (2007). Raman Spectroscopy of Biological Tissues. Appl. Spectrosc. Rev..

[37] Premasiri W.R., Lee J.C., Ziegler L.D. (2012). Surface-enhanced Raman scattering of whole human blood, blood plasma, and red blood cells: Cellular processes and bioanalytical sensing. J. Phys. Chem. B.

