# Supplementary material for: Visibility-Graph Analysis of Raman Spectra in Chalazion and Lipid Dysregulation: An Exploratory Study
Source: Entropy (Basel). 2026 Sep 11;28(9):1011. doi: 10.3390/e28091011 (PMC13605477; doi:10.3390/e28091011)
Supplement: Supplementary file 1 [file entropy-28-01011-s001.zip › entropy-4460257-supplementary.pdf]

Supplementary Material

Supplementary Table S1. Raman band assignments of spectra obtained from chalazion secretion [21–25].

Peak position [cm^{-1}]	Molecular vibration	Biomolecule assignment
621	C-C twisting mode of phenylalanine	proteins
641	ν(C-S), C-C twisting	proteins
653	Tyr	proteins
664	δ(C = O - C)	fatty acids
676	δ(C = O - C)	fatty acids
690	ring breathing modes	DNA
703	cholesterol ring deformation	cholesterol, cholesterol esters
717	C–C twisting, C-N, CN2(CH3)3	lipids, proteins
735	ν(C-C), ν(C-S)	pProteins, lipids
747	DNA	DNA
753	Trp	proteins
789	O-P-O,	DNA
814	ν(C-C), ν(PO2-2)	proteins, DNA
830	Tyr	proteins
854	Tyr	proteins
879	Trp	proteins
896	(C - O - O) skeletal	proteins, lipids
911	β(CH)	fatty acids
922	N–C–C	proteins
937	β(CH)	fatty acids
953	β(CH), Trp, Val	fatty acids, proteins
1004	Phe	proteins
1033	Phe	proteins
1046	β(CH)	fatty acids
1058	ν(C-O), ν(C-N)	proteins, lipids
1065	ν(C - C), Raman skeletal optical mode B1g vibrational modes of all-trans rotomers	triacylglycerols, fatty acids
1075	ν(C - C)	triacylglycerols, fatty acids
1084	ν(C - C), Raman skeletal optical mode for gauche rotomers	cholesterol, lipids
1096	ν(C - C)	fatty acids
1128	ν(C - C), Raman skeletal optical mode Ag vibrational modes of all-trans rotomers	triacylglycerols, fatty acids, cholesterol
1157	ν (C-N), ν(C–C) in conjugated C=C molecules	proteins
1176	ν(C - C)	cholesterol
1205	HPro, Tyr	proteins
1238	amide III	proteins
1264	δ(=CH)	triacylglycerols, fatty acids, cholesterol ester
1303	τ(CH2)	triacylglycerols, fatty acids, cholesterol ester
1317	Trp, δ(C-H)	proteins
1342	νCH3, νCH2	lipids, proteins
1392	CH rocking, ν(C-N)	proteins, lipids
1420	β(CH2)	fatty acids
1446	α(CH2/CH3), δ(C-H)	triacylglycerols, fatty acids, cholesterol, cholesterol ester, proteins
1465	β(CH2/CH3)	triacylglycerols, fatty acids
1487	ν(C-N) coupled with δ(C-H)	proteins
1523	ν(C=C)	proteins, lipids
1558	indole ring (Trp)	proteins
1573	guanine, adenine, Trp	proteins
1586	Phe, HPro	proteins
1605	C=C, Ring C-C	proteins
1619	Tyr, Trp, Phe	proteins
1634	bending mode	intermolecular
1659	ν(C = C), amide I	triacylglycerols, fatty acids, proteins
1670	ν(C = C)	cholesterol, cholesterol ester
1684	ν(C=O)	lipids, proteins
1707	C=O	lipids
2850	ν s(=CH2)	triacylglycerols, fatty acids, cholesterol, cholesterol ester
2878	ν as(=CH2)	triacylglycerols, fatty acids, cholesterol ester
2910	CH stretch	lipids, proteins
2933	ν s(=CH3), out-of-plane chain	triacylglycerols, fatty acids, cholesterol, cholesterol ester
2968	ν as(=CH3)	triacylglycerols, fatty acids
3011	ν(=CH)	triacylglycerols, fatty acids, cholesterol ester

Cysteine (Cys), Phenylalanine (Phe) Tryptophan (Trp), Tyrosine (Tyr), Proline (Pro), Hydroxyproline (Hpro), sh-shoulder; ν-stretching; s- symmetric, as-asymmetric, δ-in-plane bending; γ-out-of-plane bending; The assignments were made based on references [21-25].

Supplementary Table S2. Raman band assignments of serum [24–30].

**Peak position [cm^{-1}]**	**Molecular vibration**	**Biomolecule assignment**
509	Trp	protein
545	Trp	protein
606	C-C aromatic	cholesterol
622	C-C twisting, aromatic ring	protein
644	Tyr, ν(C-S), Cys, C-C twisting, glutathione, symmetric ν(CO2), skeletal ring deformation	protein
700	CH, C-C	cholesterol, cholesterol ester
720	γ(C-H) adenine, coenzyme, Tyr, C-C stretch of Pro ring, C-S	protein, pPhospholipid, polysaccharides
744	Phe, γ(C-H)	protein, phospholipid
758	Trp	protein
828	C-O-C and C-C backbone, O-P-O, glutathione	protein, nucleic acids
852	C-O-C, ν(C-C), δ(CCH) ring Pro, Tyr	protein, saccharides
877	ν(C-C-N), C-O-C ring, ν(C-C), Trp	lipids, carbohydrate, protein
899	C-C skeletal, ν(COC)	protein, lipids
938	ν(C-C) backbone, Pro, Hpro	protein, saccharides
956	CH3, CH2 rock, PO4	protein, nucleic acids
1002	aromatic ring Phe	protein
1031	O-CH3,ν(CC) skeletal, δ(C-H), C-H in-plane bending mode of Phe, ν(C-N), δ(CH2CH3)	protein, lipids, phospholipids
1127	ν(C-N), ν(C-C), ν(C-C) skeletal of acyl backbone in lipid	protein, lipids, glucose, phospholipid
1155	ν(C-N), ν(C-C),<br>β carotene	protein, carbohydrates
1207	Trp, Tyr , Phe, ring vibration	protein
1244	ν(C-N), δ (N-H), amide III, β-sheet	protein
1271	amide III, CH2 (in-plane deformation)	lipids, protein,
1316	δ(CH2) twisting, wagging	protein, fatty acids, phospholipids
1335	CH, CH3CH2 wagging, Trp, adenine, guanine	protein, lipids, nucleic acids
1447	δ(CH2/CH3)	fatty acids, lipids, proteins, nucleic acids, phospholipid
1520	C-C, C=C	β carotene
1555	ν(C-C), C-N, NH, amide II	protein, lipids
1585	δ (C=C), Phe, riboflavin	protein
1606	δ (C=C), Phe, Tyr, δ (NH2), Cys	protein
1616	ν(C=C) , Trp, Tyr,	protein
1659	ν(C=O), ν(C=C), amide I, α-helix	lipids, phospholipids, unsaturated fatty acids, protein,
1671	ν(C=C), ν(C=O), amide I, β-sheet	protein, cholesterol
2875	ν(CH2)	fatty acids, lipids, proteins
2931	ν(CH3)	protein
3061	C–H in =C–H	unsaturated lipids
3270	ν(O–H), ν (N-H)	protein

Cysteine (Cys), Phenylalanine (Phe) Tryptophan (Trp), Tyrosine (Tyr), Proline (Pro), Hydroxyproline (Hpro), sh-shoulder; ν-stretching; δ-in-plane bending; γ-out-of-plane bending; The assignments were made based on references [24-30].
